# Reassigning the Pressure-Induced Phase Transitions of Methylammonium Lead Bromide Perovskite

**DOI:** 10.1021/jacs.2c09457

**Published:** 2022-10-19

**Authors:** Akun Liang, Javier Gonzalez-Platas, Robin Turnbull, Catalin Popescu, Ismael Fernandez-Guillen, Rafael Abargues, Pablo P. Boix, Lan-Ting Shi, Daniel Errandonea

**Affiliations:** † Departamento de Física Aplicada-ICMUV-MALTA Consolider Team, Universitat de València, c/Dr. Moliner 50, 46100 Valencia, Burjassot, Spain; ‡ Departmento de Física, Instituto Universitario de Estudios Avanzados en Física Atómica, Molecular y Fotónica (IUDEA) and MALTA Consolider Team, Universidad de La Laguna, Avda. Astrofísico Fco. Sánchez s/n, E-38206 La Laguna, Tenerife, Spain; § CELLS-ALBA Synchrotron Light Facility, Cerdanyola, 08290 Barcelona, Spain; ∥ Institut de Ciència dels Materials, Universidad de Valencia, C/J. Beltran 2, 46980 Paterna, Spain; ⊥ Institute of High Energy Physics, Chinese Academy of Sciences, Beijing 100049, China; # Spallation Neutron Source Science Center, Dongguan 523803, China

## Abstract

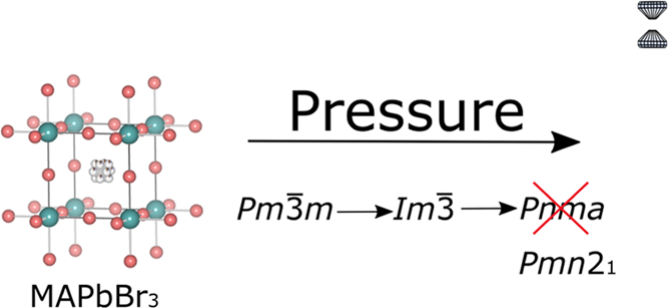

The high-pressure crystal structure evolution of CH_3_NH_3_PbBr_3_ (MAPbBr_3_) perovskite has been investigated by single-crystal X-ray diffraction and synchrotron-based powder X-ray diffraction. Single-crystal X-ray diffraction reveals that the crystal structure of MAPbBr_3_ undergoes two phase transitions following the space-group sequence: *Pm*3̅*m* → *Im*3̅ → *Pmn*2_1_, unveiling the occurrence of a nonpolar/polar transition (*Im*3̅ → *Pmn*2_1_). The transitions take place at around 0.8 and 1.8 GPa, respectively. This result contradicts the previously reported phase transition sequence: *Pm*3̅*m* → *Im*3̅ →**Pnma**. In this work, the crystal structures of each of the three phases are determined from single-crystal X-ray diffraction analysis, which is later supported by Rietveld refinement of powder X-ray diffraction patterns. The pressure dependence of the crystal lattice parameters and unit-cell volumes are determined from the two aforementioned techniques, as well as the bulk moduli for each phase. The bandgap behavior of MAPbBr_3_ has been studied up to around 4 GPa, by means of single-crystal optical absorption experiments. The evolution of the bandgap has been well explained using the pressure dependence of the Pb–Br bond distance and Pb–Br–Pb angles as determined from single-crystal X-ray diffraction experiments.

## I Introduction

Metal halide perovskites form a group of materials with the simple configuration ABX_3_ where A, B, and X are, respectively, organic parts (usually CH_3_NH_3_^+^ (MA) or NH_2_CH = NH_2_^+^ (FA)), metal cations, and halide anions (Cl^–^, Br^–^, etc.). Amongst these materials, MAPbBr_3_ and MAPbI_3_ have been found to efficiently sensitize TiO_2_ for visible-light conversion in photoelectron chemical cells, increasing the power conversion efficiency by 3.13 and 3.81%, respectively.^1^ After these results, both materials have attracted a great amount of attention. As a result of the efforts made by different research groups to study metal halide perovskites, the photovoltaic efficiency of perovskite solar cells has soared to around 25% in 2021.^2^ The tunability of bandgap energy for perovskite semiconductors is a requirement to optimize their optical properties for specific applications. For example, multijunction perovskite solar cells, where narrow-bandgap (1.1–1.2 eV) and wide-bandgap (1.7–1.8 eV) perovskites are combined, are expected to perform with an efficiency as high as 39%.^3,4^ By simply varying the ratios of I and Br in MAPb(I*_x_*Br_1–*x*_) compounds, the bandgap of hybrid perovskites can be tuned in the range of 1.6–2.3 eV;^5^ however, this can generate instabilities due to the halide segregation.^6^ Another clean method to engineer the bandgap of perovskites is by applying external pressure.^7−9^ Pressure usually shortens bond distances, changing and distorting the crystal structure, and can even induce phase transitions, thereby having a significant influence on the electronic band structure.

**Figure 1:**
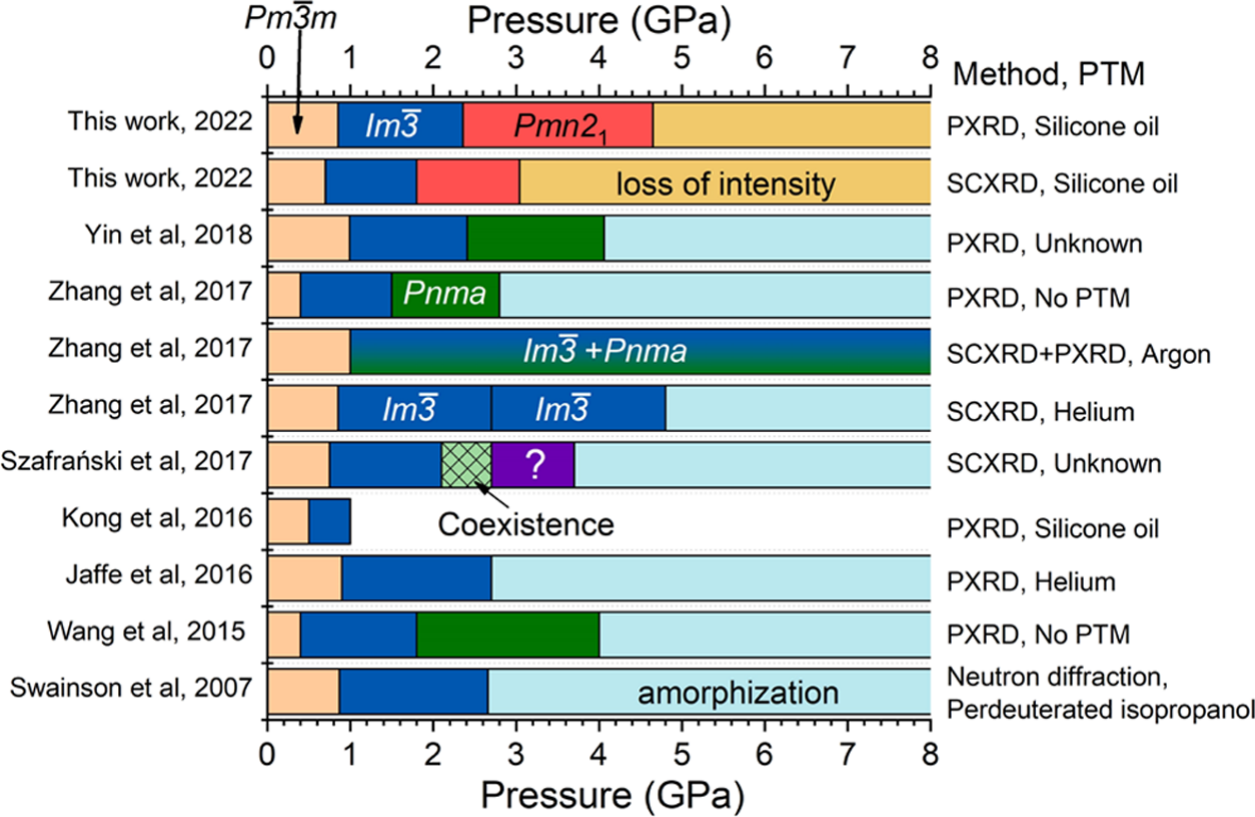
Summary of the pressure-induced phase transitions observed in MAPbBr_3_ reported in the literature, including the results reported by Swainson et al.,^10^ Wang et al.,^7^ Jaffe et al.,^12^ Kong et al.,^14^ Szafrański et al.,^8^ Zhang et al.,^15^ Yin et al.,^16^ and this work. The different crystal structures with different space groups are shown in different colors. The diffraction method, single-crystal X-ray diffraction (SCXRD), powder X-ray diffraction (PXRD), and pressure-transmitting medium (PTM) used in the studies are shown on the right-hand side.

Although several studies have been performed on the pressure-induced structural phase transitions of MAPbBr_3_, there is still much controversy in the literature as we summarize in Figure 1. In 2007, Swainson et al.^10^ investigated the pressure-induced crystal structural change of MAPbBr_3_ with neutron diffraction up to around 3 GPa at room temperature and down to around 80 K. They reported that the crystal structure transforms from space group *Pm*3̅*m* to *Im*3̅, a cubic-to-cubic phase transition, at 0.87–1.01 GPa. They also found that MAPbBr_3_ amorphized at around 2.8 GPa. In these experiments, 2-propanol-*d*_8_ (perdeuterated isopropanol) was used as pressure-transmitting medium (PTM). In 2015, Wang et al.^7^ studied the crystal structure and electronic band structure of MAPbBr_3_ under high pressure up to 34 GPa at room temperature, by powder X-ray diffraction (PXRD) in a synchrotron light source. No PTM was used in their study. Two phase transitions were observed, from *Pm*3̅*m* to *Im*3̅ at 0.4 GPa and from *Im*3̅ to **Pnma** at 1.8 GPa. In addition, amorphization was reported to take place at 4 GPa. The transition pressure was strongly affected by nonhydrostatic effects in this experiment. In addition, the assignment of space group **Pnma** was not obtained by means of indexation followed by a full-structure solution, but based on a Rietveld refinement of PXRD patterns assuming results of density-functional theory (DFT) calculations reported by Swainson et al.^10^ However, such a structure has not been experimentally found by Swainson et al.^10^ at room temperature and high pressure, being only observed at a temperature lower than 148 K at room pressure using single-crystal X-ray diffraction (SCXRD).^11^ On the other hand, PXRD results reported by Jaffe et al.^12^ also contradict the existence of a high-pressure **Pnma** structure. These studies were performed using helium as PTM, which provides hydrostatic conditions up to 12 GPa.^13^ In particular, Jaffe et al.,^12^ observed the phase transition from *Pm*3̅*m* to *Im*3̅ at 0.9 GPa and the onset of amorphization at 2.7 GPa. The crystal structures of these two cubic phases were determined by SCXRD in ambient conditions and 1.7 GPa, as well as by the Rietveld refinement of the PXRD patterns from both phases. The first phase transition and amorphization pressures are consistent with that reported by Swainson et al.^10^ Four other high-pressure studies can be found in the literature. Kong et al. carried out studies only up to 1 GPa.^14^ They only reported the phase transition from *Pm*3̅*m* to *Im*3̅ at around 0.5 GPa. In their case, silicone oil was the PTM. On the other hand, the first phase transition was observed at 0.75 GPa by Szafrański et al.^8^ In their work, the *Im*3̅ phase coexisted with an unknown phase (named phase VII in their work) in the pressure range 2.1–2.7 GPa. These authors correlate changes in the crystal structure with changes in the bandgap. They propose that the bandgap energy of MAPbBr_3_ may have a linear relationship with the Pb–Br bond length. The pressure-induced crystal structure phase transition has also been investigated by Zhang et al. with three different quasi-hydrostatic conditions (helium, argon, and no PTM).^15^ In the experiment where helium was used as the PTM, SCXRD was used to characterize the crystal structure, and the pressure-induced phase transitions from space group *Pm*3̅*m* to *Im*3̅ have been observed at 0.85 GPa, followed by an isostructural phase transition at 2.7 GPa, which was accompanied by a unit-cell volume collapse of around 4.4 Å^3^. In the second experiment where argon was used as PTM, the first phase transition was observed at 1 GPa, after that the *Im*3̅ phase coexisted with the **Pnma** phase up to the highest pressure in their work (11.9 GPa). The reason used to justify the phase coexistence was the solidification of argon at around 1.4 GPa and room temperature.^13^ In the experiment where no PTM was used, the first phase transition was observed at the lowest pressure of 0.4 GPa, in agreement with the transition pressure reported in the work from Wang et al.,^7^ followed by another pressure-induced phase transition from *Im*3̅ to **Pnma** found at 1.5 GPa. Finally, in the PXRD experiment of Yin et al.,^16^ the first phase transition (*Pm*3̅*m* to *Im*3̅), second phase transition (*Pm*3̅*m* to **Pnma**), and amorphization were located at 0.99, 2.41, and 4.06 GPa, respectively; however, the use of PTM is not reported in this work.

The crystal structures of the two low-pressure cubic phases have been unambiguously characterized by neutron diffraction, PXRD, and SCXRD, and there is an agreement, independently of the PTM used, about this fact in the literature. However, the second pressure-induced phase transition, from space group *Im*3̅ to **Pnma**, has only been observed in three papers.^7,15,16^ In two of these works, no PTM was used (i.e., experimental conditions were not hydrostatic), and in the third one, the PTM was not reported. On top of this, the crystal structure used to perform Rietveld refinements on PXRD patterns was not properly solved because it was adopted from the phase observed at low temperature and ambient pressure. The pressure-induced amorphization was indeed observed in most of the reported papers but at different pressures.

In this work, the pressure-induced crystal structure phase transitions of MAPbBr_3_ have been re-examined by SCXRD up to 5 GPa. The crystal structures have been well established up to 3 GPa, the pressure-induced phase transitions have been further confirmed by the Rietveld refinement on PXRD phases, and changes in bandgap energies have been investigated and explained. Two pressure-induced crystal structure phase transitions were found in SCXRD, PXRD, and optical experiments. The detailed crystal structure information of the three phases obtained from SCXRD will be reported, as well as the pressure-induced change in the lattice parameters and equations of state.

## II Methods

### II.I Sample Preparation

Lead(II) bromide (PbBr_2_, 98% purity, purchased from Fisher Chemical), methylammonium bromide (MABr, 98% purity, purchased from Ossila), and dimethylformamide (DMF, 99.8% purity, purchased from Sigma-Aldrich) were used as the starting materials. Lead(II) bromide and methylammonium bromide were dissolved in DMF (1 M). The solution was stirred in ambient conditions until the precursors were completely dissolved. The solution was then filtered with a 0.2 mm pore size filter, kept in a closed vial of 20 cm^3^, and heated up to 80 °C in an oil bath. The temperature ramp was set to 20 °C/h until 60 °C. Then, the solution was heated until 80 °C with a temperature ramp of 10 °C/h. Finally, it was kept at 80 °C for 24 h. Reproducible-size crystals were obtained by this method. The fine powder sample was obtained by grinding the single-crystal sample.

### II.II X-ray Diffraction

#### II.II.I High-Pressure Single-Crystal X-ray Diffraction

SCXRD has advantages over the PXRD approach because it decouples the fitting of lattice and structural parameters, leading thus to a higher resolution. In this study, SCXRD measurements were performed at room temperature using a Rigaku SuperNOVA diffractometer equipped with an EOS charge-coupled device (CCD) detector and a molybdenum radiation microsource (λ = 0.71073 Å) with a beam size of 200 μm in diameter. All measurements were processed with CrysAlisPro software.^17^ Numerical absorption corrections based on Gaussian integration over a multifaceted crystal model were applied using the ABSORB-7 program.^18^ For HP measurements, a Mini-Bragg diamond anvil cell with an opening angle of 85° and anvil culets of 500 μm diameter were used to generate the high-pressure environment. A stainless-steel gasket with a centered hole of 250 μm in diameter and 75 μm in depth was used as the gasket. Silicone oil was used as pressure-transmitting medium (PTM).^13^ Lead halide perovskite is highly soluble in polar solvents like alcohols. Therefore, nonpolar solvents like silicone oils have been used to perverse the perovskite crystal structure. The sample (a crystal of dimensions 140 μm × 70 μm × 50 μm) was placed on one of the diamond anvils (diffracting side), together with a small ruby sphere used as a pressure sensor.^19^ The crystal structure was refined for each pressure, using previous results as starting points, against *F*^2^ by full-matrix least-squares refinement implemented in the SHELXL program.^20^

#### II.II.II High-Pressure Powder X-ray Diffraction

In situ PXRD experiments were performed at the BL04-MSPD beamline of ALBA-CELLS synchrotron.^21^ A membrane Le Toullec-type diamond anvil cell (DAC), with a culet of 400 μm in diameter, was used to generate the high-pressure environment. A hole with a diameter of 200 μm drilled in the center of a preindented stainless-steel gasket with a thickness of 40 μm served as the sample chamber. As in SCXRD experiments, silicone oil was used as the PTM, and the ruby fluorescence method was used for pressure determination.^19^ The wavelength of the monochromatic X-ray beam was 0.4246 Å, and the spot size of the X-ray was 20 μm × 20 μm (full width at half-maximum). A Rayonix SX165 CCD image plate was used to collect the diffraction patterns, and the sample-to-detector distance was calibrated using a LaB_6_ standard. The collected two-dimensional diffraction images were reduced to conventional XRD patterns using DIOPTAS.^22^ The FullProf^23^ suite was used to perform Rietveld refinements.^24^

### II.III High-Pressure Optical Absorption

A membrane-type DAC was used to generate the high-pressure environment; the culet of the diamond was 400 μm. A stainless-steel gasket was first preindented to a thickness of 40 μm, and then a 200 μm diameter hole was drilled in the center, which served as a sample chamber. A single-crystal sample (90 μm × 65 μm ×10 μm in width, height, and thickness, respectively, see Figure S1 in the supporting information), together with silicone oil (PTM) and a ruby sphere (pressure gauge) was loaded in the sample chamber. The sample-in and sample-out methods were used to acquire the optical absorption spectra in a home-built optical setup, consisting of a tungsten lamp, fused silica lenses, reflecting optics objectives (15×), and a visible-near infrared spectrometer (Ocean Optics Maya 2000 pro). The light was focused on the sample and the spot size was 20 μm in diameter. The light transmitted through the sample [*I*(ω)] was normalized by the intensity of the light transmitted through the PTM [*I*(ω_0_)]. More details on the experimental setup can be found in our previous work.^25−27^

## III Results and Discussion

**Figure 2:**
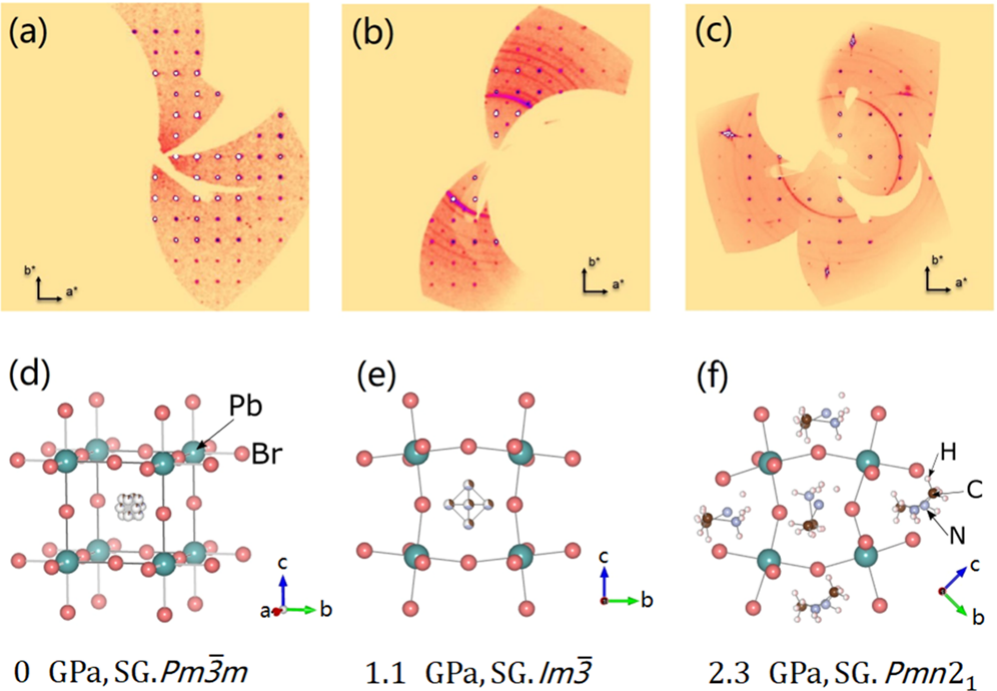
Results of high-pressure SCXRD experiments on MAPbBr_3_. (Top) Reconstructed reciprocal-space precession-images for the (*hk*0) plane in (a) ambient conditions, (b) 1.1 GPa, and (c) 2.3 GPa. They correspond to phases I, II, and III. In phases I and II, the order of rotational symmetry is 4. In phase III, it is 2. (Bottom) (d–f) show the crystal structure of MAPbBr_3_ obtained from the SCXRD data shown above. The space group (SG) of each crystal structure is shown at the bottom. The atoms are shown in different colors as indicated in the figure.

SCXRD images at different pressures are shown in Figure 2, as well as the crystal structures obtained from the experiments. Details of the data collection, refinement results and quality factors, and crystal structure information at each pressure in the experiments can be found in Table S1 of the supporting information. CIF files at each pressure can be obtained from the Cambridge Crystallographic Data Centre (CCDC). The deposition numbers of the CIF files can be found in Table S1 of the supporting information. In addition, the detailed atomic positions at ambient pressures, 1.14 GPa and 2.30 GPa are provided in Tables S2–S4 of the supporting information. In ambient conditions, MAPbBr_3_ crystallized in the cubic structure, described by space group *Pm*3̅*m*. The crystal structure determined here in ambient conditions is in agreement with that reported in all the previous studies,^7,8,10,12,14−16^ and it is schematically represented in Figure 2d. Here, we name it as phase I. In this structure, Pb atoms are bonded with six Br atoms forming a regular octahedron. The six Pb–Br bonds have a length of 2.9642 ± 0.0011 Å. The PbBr_6_ octahedra are bridged by corner-sharing Br atoms. The Pb–Br–Pb angle is 180° forming PbBr_6_ octahedra in a linear chain. The organic molecule is located at the center of the cubic structure with an important positional disorder. The SCXRD pattern collected at 1.1 GPa is different from that collected under ambient conditions (see Figure 2a,b), and we also found a change in the PXRD experiment at similar pressure, as we show later in this section. Both results support that a pressure-induced structural phase transition has taken place. Here, we name the second phase as phase II. The phase transition pressure (phase I to phase II) we found in the SCXRD experiment is 0.81 GPa, in agreement with the phase transition pressure reported in refs (8, 10, 12, 15, 16). where helium or perdeuterated isopropanol was used as PTM (Figure 1). The crystal structure of phase II determined from our SCXRD data can be described by space group *Im*3̅. It is consistent with the results reported in refs (7, 8, 10, 12, 14−16). determined from neutron diffraction, SCXRD, or PXRD. In phase II (Figure 2e), the PbBr_6_ octahedron remains regular, the six Pb–Br bonds are equal in length, and the bond distance is 2.9304 ± 0.0012 Å at 1.1 GPa. However, the Pb–Br–Pb bonds are not straight anymore, and the angle of Pb–Br–Pb is 161.4 ± 0.3°. A second pressure-induced phase transition was observed at 1.8 GPa in our SCXRD experiment. Diffraction data of the second HP phase (phase III) at 2.3 GPa are shown in Figure 2c.

The crystal structure determined is orthorhombic (Figure 2f), and the space group is *Pmn*2_1_ (no. 31). Here, we name the third phase as phase III. The crystal structure determined here from SCXRD is different from the previous results (space group **Pnma**),^7,15,16^ and it is confirmed by the Rietveld refinements of our PXRD patterns, as shown later. The unit cell in our structure (*Pmn*2_1_) is doubled in comparison with the previously proposed structure (**Pnma**). In addition, our structure provides a goodness of fit (1.084) considerably smaller than the previously proposed structure (11.247). To confirm our structure and detect possible symmetry elements not considered initially in the resolution, we have applied the procedure ADDSYM that is implemented in the PLATON program.^28^ This procedure is an improved version of the algorithm MISSYM^29,30^ based on the search for additional symmetries by evaluating the given set of coordinates. It is specially designed to detect crystal system changes, changes to the Laue class without changing the crystal system, detection of inversion centers, and translational symmetry detection. All of these tests were performed without detecting settings changes for the current cell nor the presence of an inversion center that would suggest a change from *Pmn*2_1_ to *Pmna*. Thus, we are fully confident in our structural determination. Notice that the structure in the **Pnma** space group was obtained at low temperature and used to analyze ambient temperature powder XRD experiments without making an indexation and analyzing systematic extinctions. In contrast, our structure in the *Pmn*2_1_ space group has been solved from single-crystal XRD using state-of-the-art methods, which are more robust and accurate.

Interestingly the transition from phase II to phase III involves a clear symmetry breaking into a polar space group that could potentially be important for the optoelectronic properties. We hypothesize that the polar distortion is most probably correlated with the presence of hydrogen bonding between MA and the PbBr_6_ framework, and its existence under high pressure could be related to the increase of rotational degrees of freedom of the MA group.^31^ A deeper discussion of the causes of polar distortion is beyond the scope of this work. For the crystal structure of phase III collected at 2.3 GPa (Figure 2f), the PbBr_6_ octahedra are not regular anymore, Pb is located at two different Wyckoff positions, and Br is located at eight different Wyckoff positions. The Pb–Br bond distances range from 2.859 ± 0.015 to 3.034 ± 0.015 Å, wherein the Pb–Br–Pb angle varies in the range of 142.0 ± 0.5 to 172.1 ± 0.5°. We postulate that the distortion of the PbBr_6_ octahedra after the phase transition could be caused by the enhancement of bonding between MA cations and PbBr_6_ octahedra in a similar fashion as observed in MAPbI_3_.^32^

To compare our structure (*Pmn*2_1_) with the previous **Pnma** structure, the following transformation (*b*, 2*a*, *c*) should be applied to the **Pnma** cell. The main differences are the doubling of the unit cell along the *a*-axis of the **Pnma** structure (*b*-axis of *Pmn*2_1_) and a shift of alternative (100) planes of the **Pnma** structure [(010) planes in *Pmn*2_1_]. There is also a change in the octahedral distortion of the Br in the Pb environment. In the **Pnma** structure, we have a single representative octahedron with a quadratic elongation of 1.007 and an angular variance of only 25.64 deg^2^. For the case of *Pmn*2_1_, there are two representative octahedra with a quadratic elongation of 1.012 and 1.013, respectively, so it does not present significant changes. This situation changes radically with the variance of the octahedral angles, which has values of 44.42 and 44.31 deg^2^, respectively.

At pressures higher than 3.04 GPa in the SCXRD experiment, the quality of the diffraction data quickly decreases, probably due to degradation of the monocrystal after two subsequent phase transitions, and it becomes impossible to accurately resolve the structure. In a previous study, it has been reported that the studied material undergoes a pressure-induced amorphization at 2.8 GPa.^15^ However, since the transformations between phases I–II and II–III are continuous in nature with group–subgroup relationships between the phases (*Pmn*2_1_ ⊂ *Pmmn* ⊂ **Immm** ⊂ *Im*3̅), and amorphization is usually a sign of a hindered first-order transition where a high energetic barrier prevents the formation of a new crystal phase; we do not think that the loss of intensity could be related to a gradual amorphization of MAPbBr_3_. Figure S1 in the supporting information shows pictures of the crystal up to 4.1 GPa and it does not show any visual evidence of amorphization.

**Figure 3:**
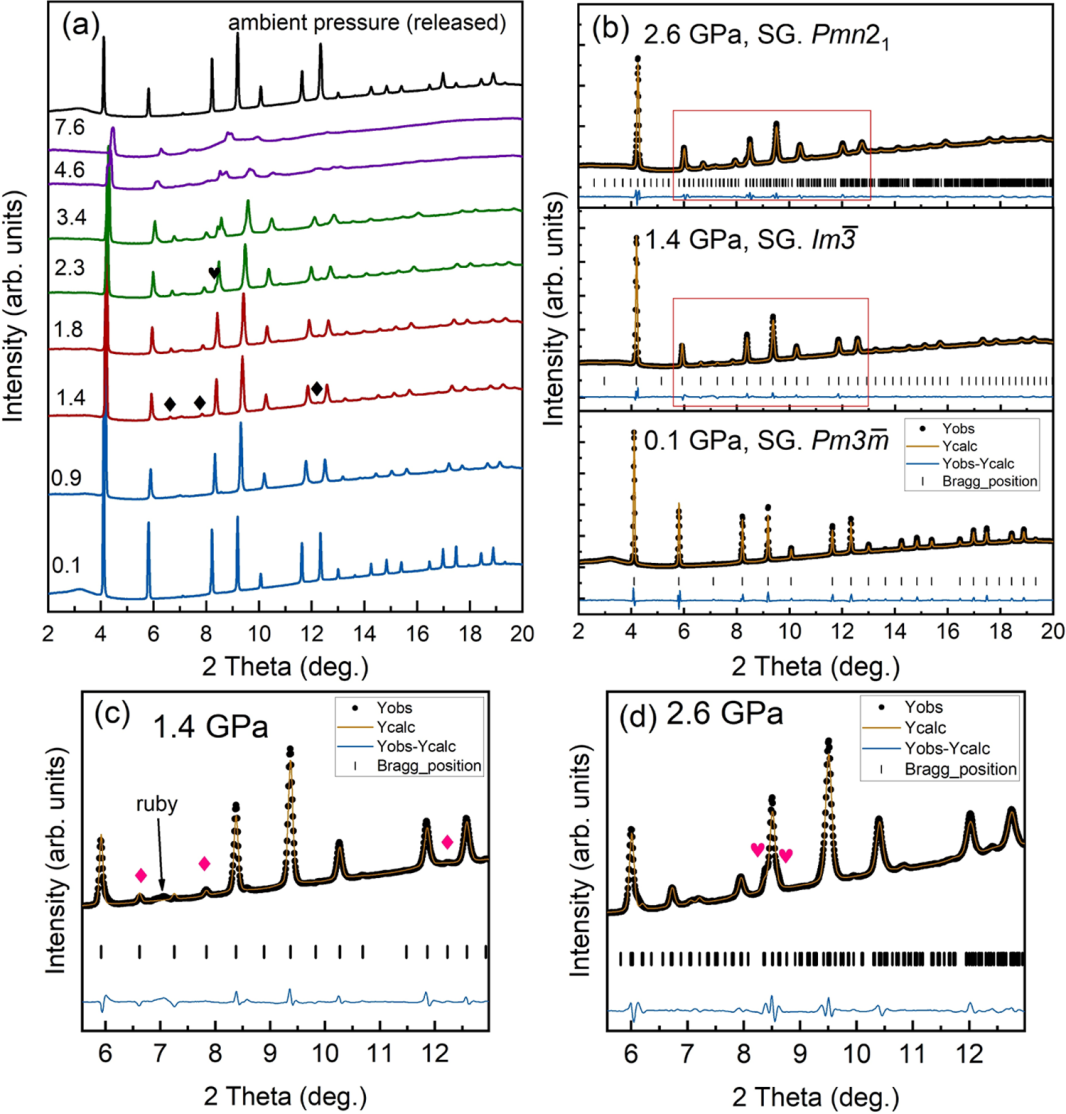
Results of high-pressure PXRD experiments on MAPbBr_3_. (a) PXRD patterns at selected pressures, and patterns from different phases are shown in different colors. Phases I, II, and III are shown in blue, red, and green, respectively. The patterns in purple show signs of a pressure-induced peak broadening and loss of intensity in the XRD patterns. Pressures are given in GPa. The black diamond and heart symbols identify the appearance of new reflections. (b) Typical Rietveld refinements at 0.1 GPa (phase I), 1.4 GPa (phase II), and 2.6 GPa (phase III). (c) and (d) Enlarged images of the areas marked by red boxes in panel (b) for experiments collected at 1.4 and 2.6 GPa, respectively. The black dots are experimental results (Yobs), the refined patterns (Ycalc) are shown in solid yellow lines, and the difference between experiments and refinements (Yobs–Ycalc) are shown in solid blue lines. The vertical ticks show the position of diffraction peaks (Bragg position). In panel (c), a peak from the ruby chip used to measure pressure is identified.

PXRD patterns of MAPbBr_3_ at selected pressures are shown in Figure 3a. At pressures lower than 0.9 GPa, they can be well refined by the ambient-pressure cubic crystal structure (phase I, space group: *Pm*3̅*m*, *R*_p_ = 1.05 and *R*_wp_ = 1.97) obtained from the SCXRD experiment. As an example, we provide in Figure 3b the Rietveld refinement at 0.1 GPa. At 1.4 GPa, there are two additional peaks located between 6 and 8° (marked by black diamonds in Figure 3a and pink diamonds in Figure 3c). Notably, the same extra peaks also have been observed in the PXRD patterns reported in refs (7, 15, 16). Another additional peak can be observed at 1.4 GPa at around 12°. This peak is too weak to be observed in Figure 3a, but it can be identified in Figure 3c. The emergence of the new peaks indicates a pressure-induced phase transition. Furthermore, the Rietveld refinement of the PXRD pattern at 1.4 GPa (Figure 3b,c) shows that all peaks can be explained by the cubic crystal structure described by space group *Im*3̅ as we determined for phase II in the SCXRD experiment (*R*_p_ = 0.60 and *R*_wp_ = 1.17). At pressures above 1.8 GPa, two extra peaks appear on either side of the peak located at around 8.2° (marked by a black heart in Figure 3a and pink hearts in Figure 3d), indicating another pressure-induced phase transition. These extra peaks have also been observed in ref (16), but in those studies, the space group of the third phase has been assigned to **Pnma** following the assignment made in ref (7). However, the structural solution made in ref (7) raises some doubts since there are some peaks at low angles (the experimentally observed peaks at around 3.2 and 6.5°) not explained by their proposed structure. Moreover, there are peaks predicted by the **Pnma** structure but not observed in the experiments. For instance, the simulation predicted a peak at around 9.5°, which is not present in experiments. In contrast, as the Rietveld refinement of the PXRD collected at 2.6 GPa (Figure 3b,d) shows (*R*_p_ = 0.61 and *R*_wp_ = 1.13), the PXRD pattern can be satisfactorily explained by the crystal structure with a space group *Pmn*2_1_ (no. 31) determined from our SCXRD experiments for phase III. In all our experiments, the new peaks (which are the sign of pressure-induced phase transitions, both phase I → phase II and phase II → phase III) are properly indexed by the crystal structure determined from SCXRD in this work (Figure 3c,d). Therefore, the Rietveld refinements of the PXRD patterns confirm the crystal structure determined from SCXRD. In addition, we also conducted a Rietveld refinement on the same PXRD data collected at 2.6 GPa by assuming the crystal structure with space group **Pnma**(33) (see Figure S2 in the supporting information), and the quality factors of the refinement are *R*_p_ = 1.80 and *R*_wp_ = 2.71 (considerably larger than in the structure here proposed, which gives *R*_p_ = 0.61 and *R*_wp_ = 1.13). It is worth noting that there are at least four peaks unindexed by the structure **Pnma**, including the extra peak appearing at around 8.5° at 2.6 GPa, which gives evidence of the pressure-induced phase II → phase III phase transition.

With further increasing pressure, at 4.6 GPa and beyond, the intensity of the diffraction peaks is reduced, most peaks become broader and most peaks for values of 2θ higher than 11° disappear (as the purple PXRD patterns show in Figure 3a). This might be caused by a gradual disordering of the crystal structure related to the partial amorphization of MAPbBr_3_, which was proposed to occur based on previous PXRD experiments.^7,12,15,16^ Such a phenomenon has only been observed in powder XRD experiments. From the present and previous studies, it cannot be concluded if it is inherent to the behavior of MAPbBr_3_ under compression or could be caused by artifacts such as grain-to-grain stresses, which could induce deformation and disorder of the crystal structure, in particular, in highly compressible materials as MAPbBr_3_.^34^

Furthermore, the pressure-induced structural changes in MAPbBr_3_ are totally reversible, as shown by the PXRD pattern collected after the pressure was released to ambient pressure (see the topmost spectra in Figure 3a). This is consistent with the reversibility found in previous studies.^7,15,16^

**Figure 4:**
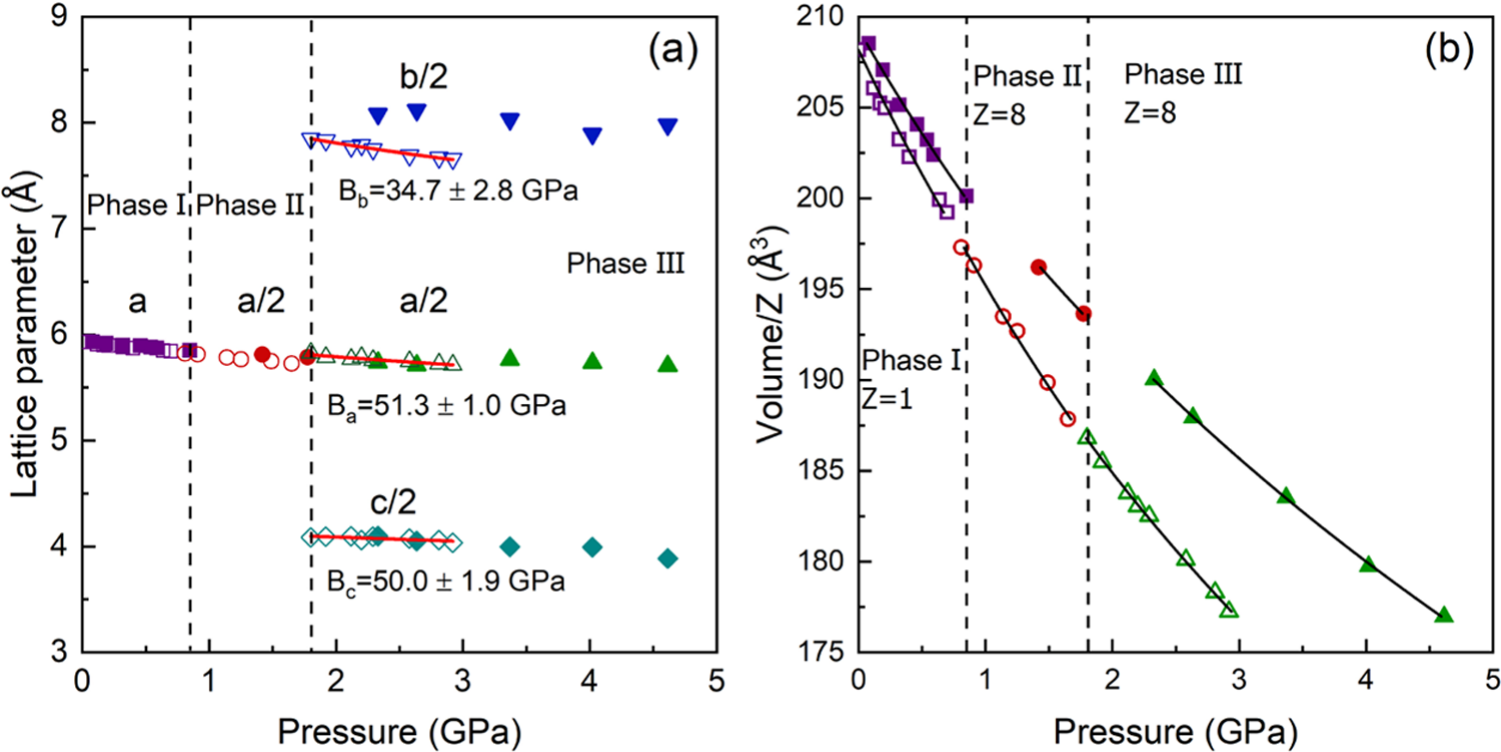
Pressure dependence of the lattice parameters and unit-cell volume of MAPbBr_3_. (a) Crystal lattice parameters obtained from PXRD (solid symbols) and SCXRD (empty symbols) as a function of pressure. The lattice parameters in phase II and III have been divided by 2 to better compare with phase I. The vertical dashed lines indicate the phase transition pressure by considering the data from both PXRD and SCXRD experiments. The red solid lines in phase III are the EOS fitting of the lattice parameter obtained from SCXRD experiments. (b) Unit-cell volume per formula unit as a function of pressure obtained from PXRD (solid symbols) and SCXRD (empty symbols). The black solid lines are the second-order Birch–Murnaghan fitting.

The lattice parameter and the unit-cell volume per formula unit as a function of pressure obtained from our experiments are plotted in Figure 4 and the data can be found in Tables S5 and S6 in the supporting information. From the structure information of the three phases summarized in Table S1 in the supporting information, it can be seen that lattice parameters from phases II and III nearly doubled the lattice parameter from phase I, and consequently, the unit-cell volume becomes approximately 8 times that of phase I. Then, for a better comparison in Figure 4, the lattice parameters from phases II and III are divided by 2 and the unit-cell volume per formula is represented. There is no observable discontinuity in the unit-cell volume at the phase transitions (Figure 4b). This observation and the fact that there is a group–subgroup relationship between the space groups of phases II and III suggests that the structural transformation could be a displacive transition. On the other hand, at the second transition, the crystal structure is elongated in one direction (*b-*axis) and shortened in the other (*c-*axis), while the third direction remains unmodified (*a-*axis). In the figure, it is shown that the lattice parameters obtained from PXRD and SCXRD show good agreement with each other.

The unit-cell volumes per formula unit for each phase have been fitted separately by second-order Birch–Murnaghan (BM) equations of state (BM-EOS) (Figure 4b).^35,36^ The second-order truncation was used to allow comparison with previous studies where a 2nd order EOS was always employed.^10,12,15^ The obtained bulk modulus and its pressure derivatives are summarized in Table 1, together with the value reported in the previous studies using different experimental methods and PTM. In this work, the bulk moduli for phases I and II obtained by fitting the unit-cell volume per formula unit (according to SCXRD) as functions of pressure are in agreement with the values reported in ref (10), where neutron diffraction was used to measure the unit-cell volume, and isopropanol was used as PTM, also in agreement with the value reported in ref (15), in which the unit-cell volume of MAPbBr_3_ is determined from the SCXRD experiment with helium as PTM. However, we did not find any sign of the pressure-induced isostructural alleged phase transition at around 2.7 GPa as in ref^15^ and the accompanied ∼4.4 Å^3^ drop in the volume. The bulk modulus for phase I from the PXRD experiment in this work is similar to the data reported in ref (12), where the probing method is PXRD and helium was used as the PTM. Unfortunately, the bulk modulus in phase II obtained from the PXRD in this work is determined from only two experimental points, and it is higher than any reported values in the literature. There is no reported experimental bulk modulus of phase III, which is described by space group **Pnma** in the previous work^7,15,16^ but unambiguously by *Pmn*2_1_ in this work. It is 13.0 and 19.1 GPa calculated from the SCXRD and PXRD experiments, respectively. There is a discrepancy in the bulk modulus determined from SCXRD and PXRD in this work, and the same phenomenon has also been observed in previous studies, which show a larger bulk modulus in PXRD than that determined from the SCXRD experiment when using helium as PTM.^12,15^ Therefore, the differences cannot be related to deviatoric stresses induced by nonhydrostatic conditions. Similar differences have been observed in other compounds, like FeVO_4_, PbCrO_4_, and BiMnO_3_^34,37,38^ being related to the existence of grain–grain stresses in powder XRD experiments. It should be noted here that the three phases are highly compressible with values of the bulk modulus comparable to that of metal–organic frameworks.^39^

**Table 1:** Summary of the Bulk Moduli (*B*_0_) for Different Phases of MAPbBr_3_^a^

phase	method	PTM	*V*_0_/*Z* (Å^3^)	*B*_0_ (GPa)	ref
*Pm*3̅*m*	ND	isopropanol	208.1 (1)	15.6 (4)	(10)
	PXRD	helium	207.8 (5)	17.6 (4)	(12)
	SCXRD	helium	∼208	12.2 (8)	(15)
	SCXRD	silicone oil	208.2 (1)	14.0 (3)	b
	PXRD	silicone oil	208.5 (1)	19.6 (8)	b
*Im*3̅	ND	isopropanol	207.8 (8)	14.1 (5)	(10)
	PXRD	helium	209.1 (1)	12.0 (1)	(12)
	SCXRD	helium	unknown	13.5 (6)	(15)
	SCXRD	helium	unknown	16.1 (9)	(15)
	SCXRD	silicone oil	208.4 (8)	12.4 (6)	b
	PXRD	silicone oil	208.5 (8)	19.2 (1)	b
*Pmn*2_1_	SCXRD	silicone oil	208.5 (5)	13.0 (3)	b
	PXRD	silicone oil	208.9 (4)	19.1 (3)	b

a “ND” means neutron diffraction, “PXRD” means powder X-ray diffraction, and “SCXRD” single-crystal X-ray diffraction. The pressure-transmitting medium (PTM) used in experiments is indicated. All results correspond to second-order Birch–Murnaghan equations of state. The zero-pressure volume per formula (*V*_0_/*Z*) is also included in this table.

b This work.

We also fitted the lattice parameters *a*, *b*, and *c* obtained from our SCXRD experiment (Figure 4a) in phase III with the 2nd order Murnaghan EOS^40^ incorporated in EoSFit7c:^41^ the linear moduli along axes *a*, *b*, and *c* are 51.3 ± 1.0, 34.7 ± 2.8, and 50.0 ± 1.9 GPa, respectively. The crystal structure in phase III shows an anisotropic behavior under compression, and the *b*-axis is the most compressible axis.

**Figure 5:**
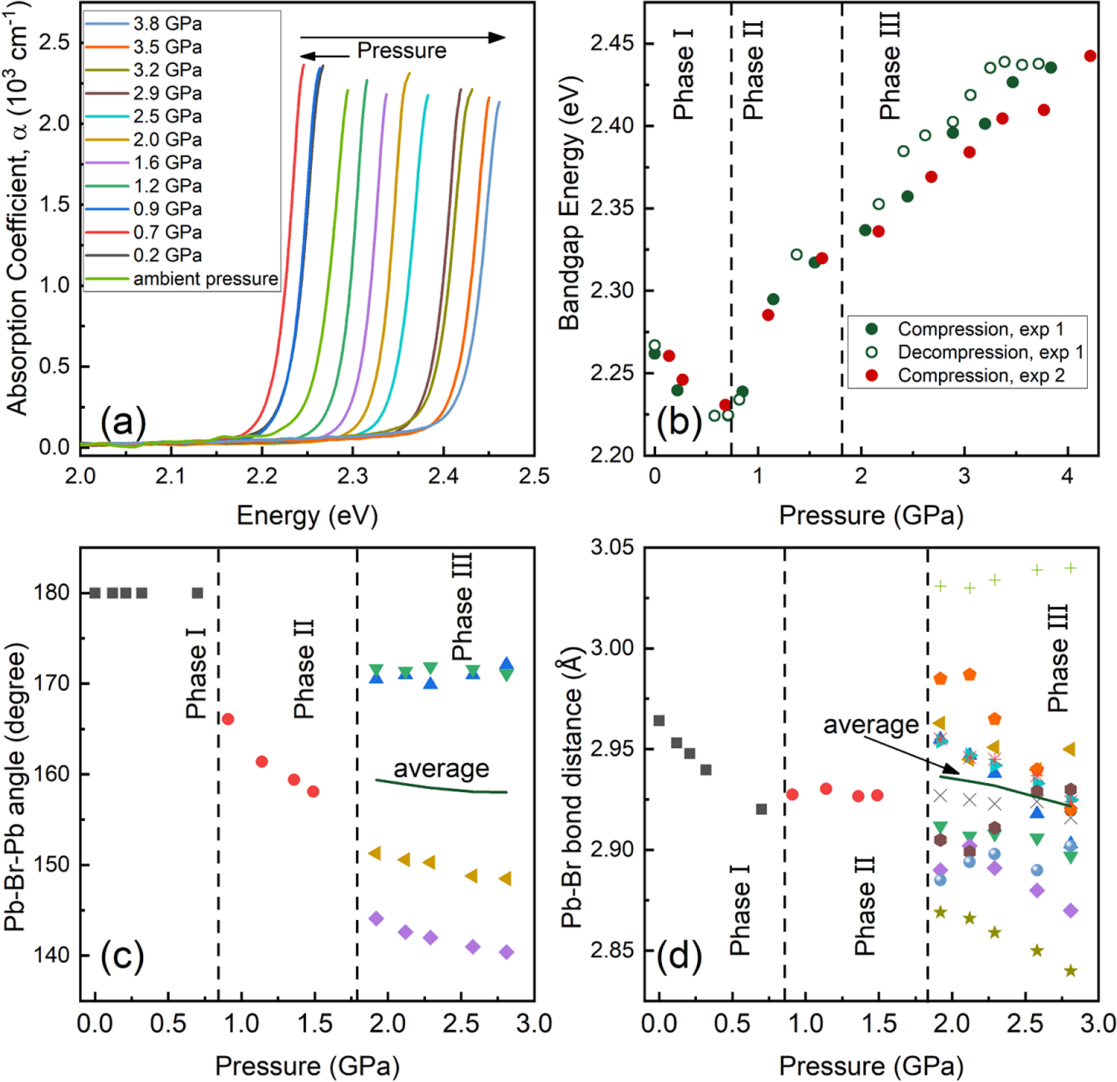
Pressure dependence of bandgap of MAPbBr_3_. (a) Optical absorption spectra of MAPbBr_3_ at selected pressures from the first experiment (exp 1). (b) Bandgap energy of MAPbBr_3_ as a function of pressure, the bandgap here at each pressure was derived from the optical absorption spectra shown in panel (a) by means of a Tauc plot. (c) Pressure dependence of Pb–Br–Pb angles and (d) Pb–Br bond distance obtained from SCXRD experiments. The vertical dashed lines indicate the phase transition pressure. In panels (c) and (d), the average Pb–Br–Pb angles and Pb–Br bond distance of phase III are shown in solid green lines.

Two independent high-pressure optical absorption experiments were performed to investigate the bandgap of MAPbBr_3_. The optical absorption spectra from the first experiment (exp 1) at selected pressure are shown in Figure 5a, and the optical image of the loading at selected pressures can be found in Figure S1 in the supporting information during both the compression and decompression processes. Changes observed are fully reversible without hysteresis. The absorption edge first shows a red shift from room pressure up to around 0.7 GPa, after that the absorption edge exhibits a blue shift under compression up to 3.8 GPa. We did not conduct any theoretical calculation on the electronic band structure of MAPbBr_3_ because of the partial occupations in phase I, but according to the previous calculations,^7,42^ the bandgap shows a direct nature. Therefore, the Tauc plot for direct bandgap materials was used to obtain the bandgap energy from the optical absorption spectra at each pressure,^43^ by extrapolating the linear fit of the high-energy part of the (α*hν*)^2^ vs *hν* plot to zero, where α, *h*, and *ν* are the absorption coefficient, Plank constant, and photon frequency, respectively. The bandgap derived from the two optical absorption experiments (exp 1 and exp 2) are in good agreement (Figure 5b). The bandgap decreases with the increasing pressure in phase I and increases in phases II and III with a different slope. The pressure-induced bandgap change is totally reversible, as the bandgap collected at the decompression process of the first experiment shows (Figure 5b). According to the previous theoretical calculations, the valence band maximum (VBM) is dominated by the Br-4p orbitals, while the conduction band minimum (CBM) is dominated by the Pb-6p orbital.^7^ Therefore, the bandgap of MAPbBr_3_ is strongly affected by the bond distance of Pb–Br and the Pb–Br–Pb angle.^25^ Furthermore, the positive linear relationship between the Pb–Br bond distance and bandgap energy of MAPbBr_3_ and MAPbI_3_ have been established in ref (8). On the other hand, the decrease of Pb–Br–Pb angle causes the opening of the bandgap energy in MAPbBr_3_.

Now, the pressure-induced bandgap change of MAPbBr_3_ can be explained by the pressure dependence of the Pb–Br bond distance and Pb–Br–Pb angle as shown in Figure 5c,d, which is obtained from SCXRD experiments. In phase I, both Pb and Br atoms are located at only one Wyckoff position (each of them), all of the Pb–Br bonds are identical and shortenend with increasing pressure, and there is no pressure-induced titling of the PbBr_6_ octahedra. Therefore, the pressure-induced narrowing of the bandgap energy is caused by the shortening of the Pb–Br bond distance under compression, which favors an increase in atomic hybridization. In phase II, the Pb–Br bond distance shows an independent behavior of pressure, the Pb–Br–Pb angle dramatically bends from 180° to around 165° and further decreases with increasing pressure, so the bandgap starts to broaden under compression. In phase III, Pb is located at two Wyckoff positions, and Br is located at eight Wyckoff positions, so there are 12 different Pb–Br bond distances and 4 different Pb–Br–Pb angles. We have calculated the average Pb–Br bond distance and Pb–Br–Pb angles as shown in Figure 5c,d. The average Pb–Br bond distance slightly decreases with increasing pressure, as well as the average Pb–Br–Pb angles. These two effects compete under compression, causing a slight increase in the bandgap energy under compression.

## IV Conclusions

In this work, we have reported the results of single-crystal X-ray diffraction (SCXRD), synchrotron-based powder X-ray diffraction (PXRD), and optical absorption experiments performed on MAPbBr_3_ perovskite under high pressure. Two pressure-induced phase transitions have been independently observed through the three different diagnostics. The crystal structures of each of the three MAPbBr_3_ phases have been determined from high-pressure SCXRD, the transition sequence is *Pm*3̅*m* → *Im*3̅ → *Pmn*2_1_, and the phase transitions occurred at 0.8 and 1.8 GPa according to both the SCXRD and PXRD data, respectively. The crystal structure determined from SCXRD has been used to perform Rietveld refinements on our PXRD patterns, explaining the experiments and supporting the crystal structure determined from SCXRD. The crystal structure in the third phase (*Pmn*2_1_) is different from that determined in previous studies (**Pnma**)^7,15,16^ where only PXRD was used and a full structural determination was not performed. Interestingly, the third phase here reported involves a clear symmetry breaking into a polar space group, which could potentially be important for the optoelectronic properties.

For each of the three phases, the pressure dependence of the lattice parameters obtained from SCXRD and PXRD, as well as the unit-cell volume per formula unit have been given. The bulk moduli have been calculated by fitting the unit-cell volume data with a second-order Birch–Murnaghan equation of state, and the results have been compared with previous studies. The bandgap change has been derived from optical absorption experiments, and it shows a narrowing behavior with increasing pressure in phase I (*Pm*3̅*m*), while a widening behavior in phases II (*Im*3̅) and III (*Pmn*2_1_) but with a different pressure dependence. There are two effects competing under compression, which results in a nonlinear pressure dependence of the bandgap energy. The pressure-induced shortening of Pb–Br bond distances causes the narrowing of the bandgap energy, while the decrease of the Pb–Br–Pb angles causes the opening of the bandgap energy. The pressure dependence of the Pb–Br bond distance and Pb–Br–Pb angles obtained from SCXRD experiments have been used to explain the bandgap energy change of MAPbBr_3_ under compression. All of the changes found in these three techniques are totally reversible.

## Supporting Information Available

Refinement results of the SCXRD experiment at each pressure; atomic position at ambient pressures, 1.14 and 2.30 GPa; and lattice parameters obtained from both SCXRD and PXRD experiments; photographs of the loading at low and high pressures; and Rietveld refinement of the PXRD spectra collected at 2.6 GPa using the **Pnma** structure (PDF)
