# Supplementary material for: Reassigning the Pressure-Induced Phase Transitions of Methylammonium Lead Bromide Perovskite
Source: J Am Chem Soc. 2022 Oct 19;144(43):20099–108. doi: 10.1021/jacs.2c09457 (PMC10388295; doi:10.1021/jacs.2c09457)
Supplement: Supplementary file 1 — ja2c09457_si_001.pdf [file ja2c09457_si_001.pdf]

Supplementary Information for

Reassigning the pressure-induced phase transitions of Methylammonium Lead Bromide Perovskite

Akun Liang, Javier Gonzalez-Platas, Robin Turnbull, Catalin Popescu, Ismael Fernandez-Guillen, Rafael Abargues, Pablo P. Boix, Lan-Ting Shi, and Daniel Errandonea

Table S1: Details of the data collections, refinement results, and structural data obtained from $MAPbBr_3$ at different pressures. Cif files have been deposited at the Cambridge Crystallographic Data Centre (CCDC).

	**0.00 GPa**	**0.12 GPa**	**0.21GPa**	**0.32 GPa**
CCDC	2194528	2210297	2210306	2210296
Formula	CH_6Br_3NPb	CH_6Br_3NPb	CH_6Br_3NPb	CH_6Br_3NPb
$D_{calc.}$/ g cm^{-3}	3.817	3.811	3.832	3.864
μ/mm^{-1}	34.524	34.908	35.097	35.392
Formula Weight	478.99	478.99	478.99	478.99
Colour	orange	orange	orange	orange
Shape	prism	prism	prism	prism
Size/mm^3	0.14×0.07×0.05	0.12×0.05×0.04	0.12x0.05x0.04	0.12×0.05×0.04
T/K	293(2)	293(2)	293(2)	293(2)
Crystal System	cubic	cubic	cubic	cubic
Flack parameter	-	-	-	-
Space Group	$Pm\bar{3}m$	$Pm\bar{3}m$	$Pm\bar{3}m$	$Pm\bar{3}m$
a/Å	5.9284(2)	5.9065(4)	5.8959(5)	5.8795(4)
b/Å	5.9284(2)	5.9065(4)	5.8959(5)	5.8795(4)
c/Å	5.9284(2)	5.9065(4)	5.8959(5)	5.8795(4)
α/°	90	90	90	90
β/°	90	90	90	90
γ/°	90	90	90	90
V/Å^3	208.36(2)	206.06(4)	204.95(5)	203.24(4)
Z	1	1	1	1
Wavelength/Å	0.71073	0.71073	0.71073	0.71073
Radiation type	Mo K_α	Mo K_α	Mo K_α	Mo K_α
Θ_{min}/°	3.437	4.881	4.890	4.903
Θ_{max}/°	26.135	26.239	26.290	26.369
Measured Refl.	513	198	199	186
Independent Refl.	513	198	199	186
Refl's I≥2 σ(I)	68	62	61	60
R_{int}	0.0376	0.0302	0.0245	0.0238
Parameters	12	7	7	7
Restraints	0	0	0	0
Largest Peak	0.584	0.84	1.06	0.86
Deepest Hole	-0.528	-0.85	-0.85	-1.03
GooF	1.106	1.227	1.193	1.248
wR_2 (all data)	0.0295	0.0633	0.0641	0.0730
wR_2	0.0295	0.0628	0.0640	0.0728
R_1 (all data)	0.0141	0.0250	0.0275	0.0296
R_1	0.0141	0.0250	0.0263	0.0282

	**0.70 GPa**	**0.91 GPa**	**1.14GPa**	**1.36 GPa**
CCDC	2210299	2210301	2194529	2210298
Formula	CH_6Br_3NPb	CH_6Br_3NPb	CH_6Br_3NPb	CH_6Br_3NPb
$D_{calc.}$/ g cm^{-3}	3.942	4.000	4.059	4.110
μ/mm^{-1}	36.107	36.641	37.174	37.648
Formula Weight	478.99	478.99	478.99	478.99
Colour	orange	orange	light orange	light orange
Shape	prism	prism	prism	prism
Size/mm^3	0.12×0.05×0.04	0.12×0.05×0.04	0.12x0.05x0.04	0.12×0.05×0.04
T/K	293(2)	293(2)	293(2)	293(2)
Crystal System	cubic	cubic	cubic	cubic
Flack parameter	-	-	-	-
Space Group	$Pm\overline{3}m$	$Im\overline{3}$	$Im\overline{3}$	$Im\overline{3}$
a/Å	5.8407(7)	11.6238 (9)	11.5680(9)	11.5192(7)
b/Å	5.8407(7)	11.6238 (9)	11.5680(9)	11.5192(7)
c/Å	5.8407(7)	11.6238 (9)	11.5680(9)	11.5192(7)
α/°	90	90	90	90
β/°	90	90	90	90
γ/°	90	90	90	90
V/Å^3	199.22(7)	1570.5 (4)	1548.0(3)	1528.5(3)
Z	1	8	8	8
Wavelength/Å	0.71073	0.71073	0.71073	0.71073
Radiation type	Mo K_α	Mo K_α	Mo K_α	Mo K_α
Θ_{min}/°	3.488	2.478	2.490	2.501
Θ_{max}/°	26.293	26.298	26.298	26.281
Measured Refl.	186	981	879	788
Independent Refl.	186	981	876	788
Refl's I≥2 σ(I)	62	159	162	174
R_{int}	0.0354	0.0445	0.0641	0.0506
Parameters	7	13	13	13
Restraints	0	0	0	0
Largest Peak	0.96	1.20	2.68	1.64
Deepest Hole	-1.50	-3.43	-8.43	-4.40
GooF	1.223	1.089	1.169	1.162
wR_2 (all data)	0.0831	0.0967	0.1966	0.1932
wR_2	0.0827	0.0815	0.1696	0.1603
R_1 (all data)	0.0360	0.0976	0.1596	0.1535
R_1	0.0349	0.0494	0.0957	0.0904

	**1.49 GPa**	**1.92GPa**	**2.12 GPa**	**2.30 GPa**
CCDC	2210303	2210305	2210302	2194530
Formula	CH_6Br_3NPb	CH_6Br_3NPb	CH_6Br_3NPb	CH_6Br_3NPb
$D_{calc.}$/ g cm^{-3}	4.136	4.288	4.328	4.358
μ/mm^{-1}	37.886	38.782	39.144	39.412
Formula Weight	478.99	478.99	478.99	478.99
Colour	light orange	light orange	light orange	light orange
Shape	prism	prism	prism	prism
Size/mm^3	0.12×0.05×0.04	0.12x0.05x0.04	0.12×0.05×0.04	0.12×0.05×0.04
T/K	293(2)	293(2)	293(2)	293(2)
Crystal System	cubic	orthorhombic	orthorhombic	orthorhombic
Flack parameter	-	0.31(10)	0.44(12)	0.45(14)
Space Group	$Im\bar{3}$	$Pmn2_1$	$Pmn2_1$	$Pmn2_1$
a/Å	11.4950(14)	11.5687(18)	11.538(2)	11.515(2)
b/Å	11.4950(14)	15.663(8)	15.549(8)	15.496(7)
c/Å	11.4950(14)	8.1891(12)	8.1949(13)	8.1831(12)
α/°	90	90	90	90
β/°	90	90	90	90
γ/°	90	90	90	90
V/Å^3	1518.9(6)	1483.9(8)	1470.2(8)	1460.2(8)
Z	8	8	8	8
Wavelength/Å	0.71073	0.71073	0.71073	0.71073
Radiation type	Mo K_α	Mo K_α	Mo K_α	Mo K_α
Θ_{min}/°	2.506	2.189	2.198	2.203
Θ_{max}/°	26.341	23.254	23.243	23.249
Measured Refl.	852	2580	2089	1999
Independent Refl.	848	2466	1998	1912
Refl's I≥2 σ(I)	182	883	841	802
R_{int}	0.0335	0.0253	0.0223	0.0255
Parameters	13	111	111	108
Restraints	0	3	3	3
Largest Peak	2.14	1.60	2.00	2.43
Deepest Hole	-5.29	-1.66	-2.16	-2.90
GooF	1.193	1.085	1.104	1.093
wR_2 (all data)	0.2187	0.1726	0.2069	0.2706
wR_2	0.1934	0.1527	0.1824	0.2375
R_1 (all data)	0.1487	0.0765	0.0908	0.1158
R_1	0.1028	0.0557	0.0673	0.0884

	**2.58 GPa**	**2.81GPa**
CCDC	2210308	2210304
Formula	CH_6Br_3NPb	CH_6Br_3NPb
$D_{calc.}$/ g cm^{-3}	4.416	4.461
μ/mm^{-1}	39.941	40.343
Formula Weight	478.99	478.99
Colour	light orange	light orange
Shape	prism	prism
Size/mm^3	0.12×0.05×0.04	0.12x0.05x0.04
T/K	293(2)	293(2)
Crystal System	orthorhombic	orthorhombic
Flack parameter	0.51(15)	0.39(19)
Space Group	$Pmn2_1$	$Pmn2_1$
a/Å	11.493(2)	11.447(3)
b/Å	15.383(9)	15.340(10)
c/Å	8.1493(14)	8.1236 (16)
α/°	90	90
β/°	90	90
γ/°	90	90
V/Å^3	1440.8 (9)	1426.5(10)
Z	8	8
Wavelength/Å	0.71073	0.71073
Radiation type	Mo K_α	Mo K_α
Θ_{min}/°	2.212	2.220
Θ_{max}/°	23.253	23.249
Measured Refl.	1926	1830
Independent Refl.	1841	1752
Refl's I≥2 σ(I)	747	693
R_{int}	0.0263	0.0309
Parameters	104	101
Restraints	3	3
Largest Peak	2.71	2.64
Deepest Hole	-2.77	-3.32
GooF	1.096	1.053
wR_2 (all data)	0.2877	0.3092
wR_2	0.2539	0.2626
R_1 (all data)	0.1231	0.1459
R_1	0.0919	0.1012

Table S2. Atomic positions of $MAPbBr_3$ at ambient condition (Phase I, Space group: $Pm\bar{3}m$) determined from the SCXRD experiment in this work.

Atom	Wyckoff	x	y	z	occupation
Pb1	1b	0.5000	0.5000	0.5000	1.000
Br1	3c	0.5000	0.0000	0.5000	1.000
C1	6e	0.0000	0.0000	-0.1190	0.167
N1	6e	0.0000	0.0000	-0.1190	0.021

Table S3. Atomic positions of $MAPbBr_3$ at 1.14 GPa (Phase II, Space group: $Im\bar{3}$) determined from the SCXRD experiment in this work.

Atom	Wyckoff	x	y	z	occupation
Pb1	8c	0.2500	0.2500	0.2500	1.000
Br1	24g	0.5000	0.2182	0.2865	1.000
C1	12e	0.5000	0.4520	0.0000	0.333
N1	12e	0.5000	0.4520	0.0000	0.333
C2	12d	0.5000	0.3990	0.5000	0.333
N2	12d	0.5000	0.3990	0.5000	0.333

Table S4. Atomic positions of $MAPbBr_3$ at 2.30 GPa (Phase III, Space group: $Pmn2_1$) determined from the SCXRD experiment in this work.

Atom	Wyckoff	*x*	*y*	*z*	occupation
Pb1	4b	-0.2484	-0.3714	0.6651	1.000
Pb2	4b	-0.2468	-0.1266	0.1816	1.000
Br1	4b	-0.2813	-0.2871	0.3469	1.000
Br2	2a	-0.5000	-0.3871	0.6750	1.000
Br3	4b	-0.2660	-0.4716	0.9621	1.000
Br4	2a	0.0000	-0.3311	0.6300	1.000
Br5	4b	-0.2790	-0.2184	0.8670	1.000
Br6	4b	-0.2518	-0.0438	0.4990	1.000
Br7	2a	-0.5000	-0.1141	0.1899	1.000
Br8	2a	0.0000	-0.1625	0.2470	1.000
N1	2a	-0.5000	0.1180	0.1560	1.000
N2	2a	-0.5000	-0.4370	0.1960	1.000
N3	2a	-0.5000	-0.1040	0.7320	1.000
N4	2a	0.0000	-0.3880	0.2000	1.000
C1	2a	-0.5000	0.1130	0.3360	1.000
C2	2a	-0.5000	-0.3510	0.0700	1.000
C3	2a	-0.5000	-0.1630	0.6000	1.000
C4	2a	0.0000	-0.3600	0.0400	1.000
H1	4b	-0.5170	0.1722	0.1263	0.500
H2	4b	-0.5529	0.0825	0.1160	0.500
H3	4b	-0.4302	0.1042	0.1185	0.500
H4	4b	-0.4477	0.0680	0.3703	0.500
H5	4b	-0.5770	0.0997	0.3739	0.500
H6	4b	-0.4753	0.1669	0.3816	0.500
H7	4b	-0.5531	-0.4288	0.2740	0.500
H8	4b	-0.4302	-0.4421	0.2420	0.500
H9	4b	-0.5167	-0.4842	0.1401	0.500
H10	4b	-0.5714	-0.3201	0.0823	0.500
H11	4b	-0.4927	-0.3710	-0.0408	0.500
H12	4b	-0.4359	-0.3144	0.0962	0.500
H13	4b	-0.5635	-0.0544	0.7659	0.500
H14	4b	-0.5013	-0.1126	0.8726	1.000
H15	4b	-0.5334	-0.2164	0.6335	0.500
H16	4b	-0.4217	-0.1719	0.5635	0.500
H17	4b	-0.5449	-0.1388	0.5115	0.500
H18	4b	0.0646	-0.4348	0.3162	0.500
H19	4b	-0.0286	-0.3815	0.3693	0.500
H20	4b	0.0709	-0.3310	0.0138	0.500
H21	4b	-0.0648	-0.3235	0.0183	0.500
H22	4b	-0.0061	-0.4106	-0.0332	0.500

Table S5. Lattice parameter and unit-cell volume at high pressure obtained from two separated single-crystal X-ray diffraction experiments.

Single-crystal X-ray diffraction					
Phase	Pressure (GPa)	a (Å)	b (Å)	c (Å)	Volume ($Å^3$)
Phase I ($Pm\bar{3}m$)	0.00	5.9266(2)	–	–	208.17(2)
	0.12	5.9065(4)	–	–	206.06(4)
	0.17	5.8985(4)	–	–	205.22(4)
	0.21	5.8959(5)	–	–	204.95(5)
	0.32	5.8795(4)	–	–	203.24(4)
	0.40	5.8701(2)	–	–	202.27(2)
	0.64	5.8472(2)	–	–	199.91(2)
	0.70	5.8404(7)	–	–	199.22(7)
Phase II ($Im\bar{3}$)	0.81	11.6431(2)	–	–	1578.36(8)
	0.91	11.6238(9)	–	–	1570.5(9)
	1.14	11.5680(9)	–	–	1548.0(3)
	1.25	11.5320(5)	–	–	1533.6(2)
	1.49	11.4950(14)	–	–	1518.9(6)
	1.65	11.454(2)	–	–	1502.7(9)
Phase III ($Pmn2_1$)	1.80	11.6489(2)	15.6940(5)	8.1743(12)	1494.4(3)
	1.92	11.5687(18)	15.663(8)	8.1891(12)	1483.9(8)
	2.12	11.538(2)	15.549(8)	8.1949(13)	1470.2(8)
	2.20	11.5729(2)	15.5770(6)	8.1236(12)	1464.5(3)
	2.29	11.515(2)	15.496(7)	8.1831(12)	1460.2(8)
	2.58	11.493(2)	15.383(9)	8.1493(14)	1440.8(9)
	2.81	11.447(3)	15.340(10)	8.1236(16)	1426.5(10)
	2.92	11.4290(3)	15.3180(8)	8.0680(11)	1412.5(3)

Table S6. Lattice parameter and unit-cell volume at high pressure obtained from powder X-ray diffraction experiments.

Powder X-ray diffraction					
Phase	Pressure (GPa)	a (Å)	b (Å)	c (Å)	Volume ($Å^3$)
Phase I ($Pm\bar{3}m$)	0.08	5.9301(1)	–	–	208.534(6)
	0.19	5.9161(1)	–	–	207.064(6)
	0.32	5.8979(1)	–	–	205.154(7)
	0.46	5.8942(1)	–	–	204.773(7)
	0.54	5.8793(1)	–	–	203.223(7)
	0.59	5.8714(1)	–	–	202.406(8)
	0.85	5.8493(1)	–	–	200.130(9)
Phase II ($Im\bar{3}$)	1.42	11.6212(3)	–	–	1569.63(8)
	1.78	11.5710(4)	–	–	1549.20(9)
Phase III ($Pmn2_1$)	2.33	11.477(2)	16.164(9)	8.200(5)	1520.3(12)
	2.64	11.425(2)	16.235(13)	8.105(6)	1503.5(17)
	3.37	11.522(2)	16.060(14)	7.995(7)	1479.5(18)
	4.02	11.466(2)	15.782(7)	7.990(4)	1445.9(9)
	4.62	11.412(3)	15.961(13)	7.774(7)	1415.8(17)

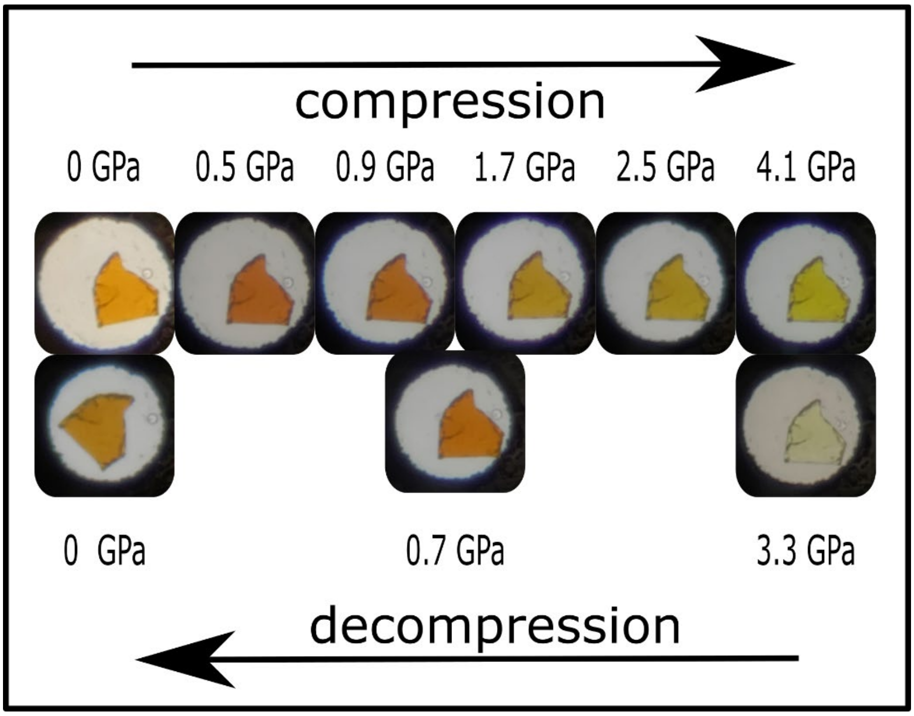

FIG. S1. Photographs of the sample loaded in the diamond-anvil cell at selected pressures.

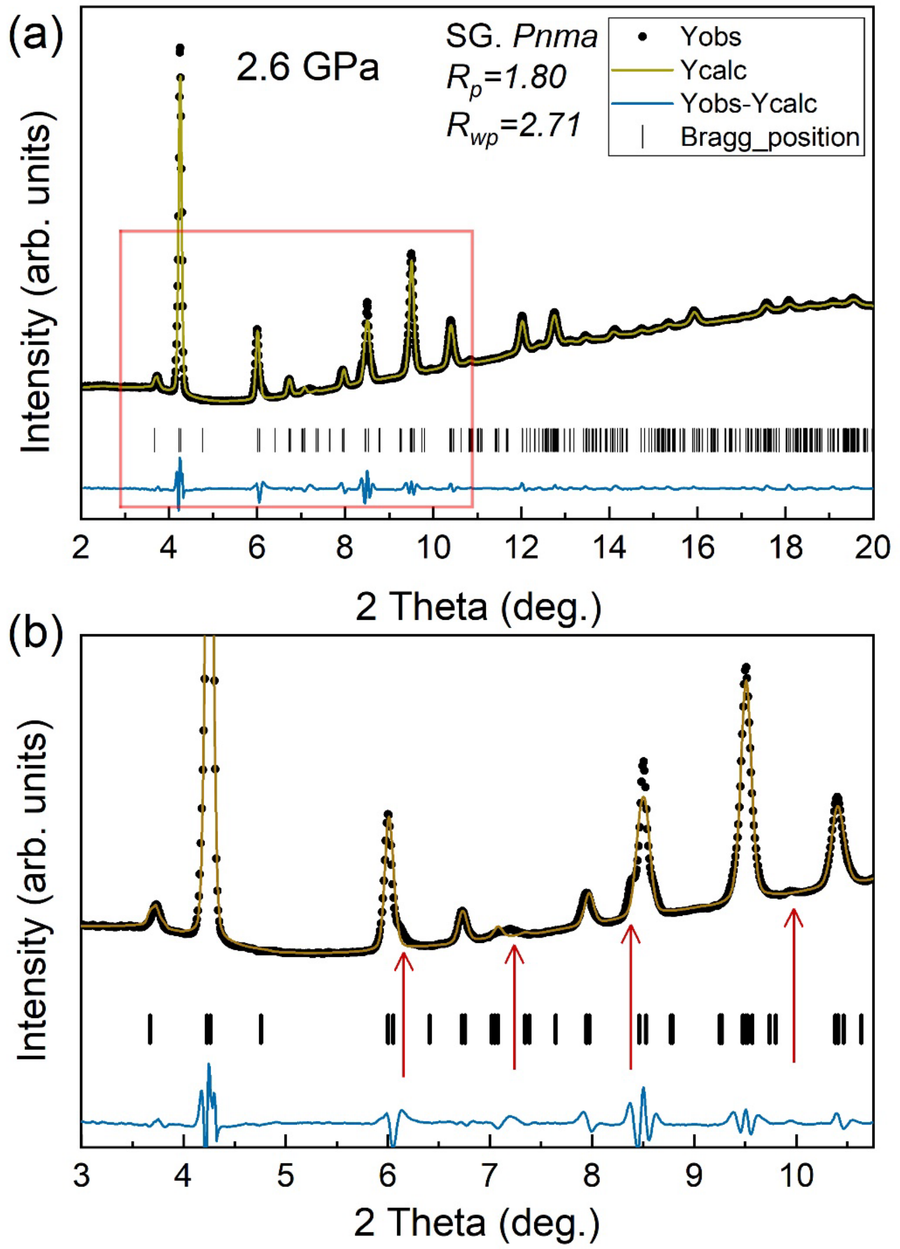

FIG. S2. (a) Rietveld refinement of the powder X-ray diffraction spectra at 2.6 GPa, the structure reported at 120 K with the space group of *Pnma* (No. 62) was used as the started structure.[1] The quality of the refinement is R_p= 1.80 and R_{wp}=2.71. (b) The enlarged spectra of the area marked by the red box in (a). Here the red vertical arrow indicted the unindexed peaks in the refinement.

References

(1) López, C. A.; Martínez-Huerta, M. V.; Alvarez-Galván, M. C.; Kayser, P.; Gant, P.; Castellanos-Gomez, A.; Fernández-Díaz, M. T.; Fauth, F.; Alonso, J. A. Elucidating the Methylammonium (MA) Conformation in $MAPbBr_3$ Perovskite with Application in Solar Cells. *Inorg. Chem.* **2017**, *56* (22), 14214–14219.
